# Automation of Cranial Nerve Tractography by Filtering Tractograms for Skull Base Surgery

**DOI:** 10.3389/fnimg.2022.838483

**Published:** 2022-03-29

**Authors:** Méghane Decroocq, Morgane Des Ligneris, Titouan Poquillon, Maxime Vincent, Manon Aubert, Timothée Jacquesson, Carole Frindel

**Affiliations:** ^1^Univ Lyon, INSA-Lyon, Université Claude Bernard Lyon 1, UJM-Saint Etienne, CNRS, INSERM, CREATIS UMR 5220, Lyon, France; ^2^Skull Base Multi-Disciplinary Unit, Neurological Hospital Pierre Wertheimer, Hospices Civils de Lyon, Lyon, France

**Keywords:** diffusion MR imaging, tractography, filtering accuracy, entropy, cranial nerve

## Abstract

Fiber tractography enables the *in vivo* reconstruction of white matter fibers in 3 dimensions using data collected by diffusion tensor imaging, thereby helping to understand functional neuroanatomy. In a pre-operative context, it provides essential information on the trajectory of fiber bundles of medical interest, such as cranial nerves. However, the optimization of tractography parameters is a time-consuming process and requires expert neuroanatomical knowledge, making the use of tractography difficult in clinical routine. Tractogram filtering is a method used to isolate the most relevant fibers. In this work, we propose to use filtering as a post-processing of tractography to avoid the manual optimization of tracking parameters and therefore making a step forward automation of tractography. To question the feasibility of automated tractography of cranial nerves, we perform an analysis of main cranial nerves on a series of patients with skull base tumors. A quantitative evaluation of the filtering performance of two state-of-the-art and a new entropy-based methods is carried out on the basis of reference tractograms produced by experts. Our approach proves to be more stable in the selection of the optimal filtering threshold and turns out to be interesting in terms of computational time complexity.

## 1. Introduction

Diffusion magnetic resonance imaging (MRI) allows to study the tissue microstructure *in vivo* non-invasively by detecting the movement of water molecules to generate specific contrast (Le Bihan and Johansen-Berg, 2012). This method has been widely used in the white matter of the brain, where the organization of axons is known to be consistent at the millimeter scale. Using diffusion MR images in a number of directions and suitable statistical models, the orientations of axonal bundles can be estimated (Tournier et al., 2011). These properties are then used to infer the structure of human brain tissue *in vivo*. From the tissue local orientations, it is possible to reconstruct a neural bundle by iteratively tracing these orientations until a termination criterion is reached. This process named “tractography” can be repeated from different starting points in a region of interest to produce an estimate of the structural connections of this region—referred to as a “tractogram” (Jeurissen et al., 2019). The biological precision of these reconstructions is however limited by the reconstruction mechanisms itself. The choice of the tractography algorithm, its settings and the tracing of local diffusion orientations can introduce biases in the tractograms which move them away from the underlying biological reality (Rheault et al., 2020).

Surgery of skull base tumors remains a challenge because it requires surgical approaches sparing the nervous tissue while allowing access to deep tumors in a complex anatomical environment made up of numerous nerves and cranial vessels (Samii and Gerganov, 2011). While cerebrovascular supply can be adequately assessed by MRI or angiography, the trajectory of the cranial nerves from the brainstem through the skull base and around tumors is not still achievable in routine clinical practice. Classic high-resolution T1 or T2 MRI sequences only allow visualization of the cisternal segment of the largest cranial nerves under normal conditions (Yousry et al., 2000). In this context, diffusion MRI and reconstruction by tractography of the path of cranial nerves displaced by tumors could be helpful for surgical planning, as attested by recent studies examining the trajectory of facial nerves in vestibular schwannomas surgery (Borkar et al., 2016; Song et al., 2016; Jacquesson et al., 2018a). Nonetheless, tractography involves a complex multi-step processing pipeline and is still difficult to apply to small-scale structures such as cranial nerves (Jacquesson et al., 2018b).

In order to extract the bundle of nerve fibers of interest, regions of interest (ROI) have to be manually delineated to initialize the tractography process. However, the design of ROI is time consuming and requires expert neuroanatomical knowledge. Besides, tractography parameters such as the minimum length of fibers, maximum angle, or fractional anisotropy (FA) threshold—used as a criterion for stopping the tractography process—also require a manual adjustment to perform an accurate reconstruction of the bundle. In this context, the automation of cranial nerve tractography arises from a growing need to save time and reduce its dependence on the user so that it can be applied systematically in clinical practice. A few studies compared fully automated tractography to user-driven tractography. The approaches chosen to automate the process mainly focus on ROI placement. In those studies, the imaging volumes were registered so that a standardized set of ROIs can be applied (Zhang et al., 2008, 2010). In their work, Nucifora et al. (2012) combined the use of an atlas-based set of ROI with post-processing treatment to filter the implausible “false” fibers. The results of this study suggest that filtering can help compensate for non-optimal ROIs and tracking parameters. In the case of cranial nerves, we believe that limitations related to the small size of the nerve fiber bundle and pathological displacement could be overcome by using filtering to discriminate nerve fibers from other structures selected involuntarily.

A number of tractogram filtering methods were proposed in the literature (Jörgens et al., 2021). The majority of approaches involve selecting the most relevant fibers based on a quality measure. This quality metric can be defined by the mean values of a diffusion metric along the fiber path (Yeh et al., 2021). This diffusion metric can be obtained from the diffusion tensor model (e.g., FA, axial, and radial diffusivities) (Everts et al., 2009), or from more sophisticated models such as constrained spherical deconvolution (CSD) (Smith et al., 2013, 2015) or other methods combining multiple approaches (Jörgens et al., 2021). Once the quality measure is defined, one way to filter tractograms consists of applying thresholds on the quality measure to filter out weak connections.

In this article, we propose to compare the performances of different filtering methods and evaluate their contribution in the context of the automation of cranial nerves tractography. To this end, a set of non-optimal tractograms is generated for five cranial nerves on a series of patients, by applying different transformations to the optimal ROI designed by the neurosurgeon, and varying the most sensitive parameters for the reconstruction of the tractograms. The non-optimal tractograms are filtered by two state-of-the-art approaches (Everts et al., 2009; Smith et al., 2013) and a new entropy-based method we propose. The non-optimal tractograms are compared to the ground truth tractograms produced by a neurosurgeon, before and after filtering. A quality metric based on Sørensen-Dice coefficient allows for a quantitative evaluation of the ability of the different filtering algorithms to isolate the cranial nerves in unfavorable cases.

## 2. Material

### 2.1. Cranial Nerves

Five cranial nerves or nerve groups were considered: the optic nerve (Chiasma); the oculomotor nerve (III); the trigeminal nerve (V); the acoustic facial bundle including nerves VII–VIII (AFB); and the lower nerves bundle including nerves IX, X, XI (LN). Their mean cisternal diameter was estimated (see Table 1) according to the known anatomy (Rhoton, 1979; Joo et al., 2014; Yoshino et al., 2016). The direction of their cisternal segment was oblique anteriorly and laterally for the optic and oculomotor nerves (Chiasma and III); straight anterior for the trigeminal nerve (V); straight lateral for the AFB bundle; and oblique laterally, anteriorly, and inferiorly for the LN bundle.

**Table 1:** Estimated diameter of the studied nerves from the literature.

**Nerve definition**	**Optic**	**Oculomotor**	**Trigeminal**	**Acoustic-facial**	**Lower**
Nerve abbreviation	Chiasma	III	V	AFN	LN
Diameter (*mm*)	10	5	7	3	2

### 2.2. Patients

Patient data used in this work are based on the study carried out between December 2015 and December 2017 in Jacquesson et al. (2018a) (IRB Number 2015-A01113-46). Inclusion criteria were as follows: skull base tumor; at least two cranial nerves in contact with the tumor; legal capacity; consent provided after fair information; 3T MRI data with diffusion MRI (dMRI) acquisition. Exclusion criteria were as follows: MR contraindications. Eight patients showing a variability in the tumor location, size, and nerves in contact with the tumor were selected for our study.

### 2.3. MRI Acquisition

MR images were obtained using a 3T MRI Ingenia machine (Philips Medical Systems, Beth, The Netherlands) with a 32-channel head coil. Five sequences were acquired: post-contrast T1-weighted; high resolution and steady state T2-weighted; time of flight (TOF); diffusion; and AP-PA sequences (pair of *b* = 0 images with opposed phase encoding direction, Andersson et al., 2003). T1 post-contrast weighted sequence was used to manually segment the tumors and T2 steady state sequence as an anatomical reference. Diffusion images were acquired with following settings: TR = 3,956 ms; TE = 102 ms; *b*-value = 1,000 s.mm^−^2; 32 directions; acquired voxel size = 2 mm isotropic; field of view: 224 mm; slice thickness = 2 mm; no slice gap; single-shot spin-echo sequence; 26 slices; and scan time 9 min 52 s. The limits of the acquisition box were the optic tracts superiorly and the foramen magnum inferiorly. Distortions were corrected using the top-up and eddy tools of the FMRIB software library (FSL) software (Smith et al., 2004).

### 2.4. Ground Truth Tractography

The tractography of the cranial nerves was carried out using the MRtrix3 software (Tournier et al., 2019). A brain mask was drawn to include the whole brainstem, the cisterns of cerebrospinal fluid, the skull base, and the orbits. A spherical constrained deconvolution (6 spherical harmonic terms) has been used to create a map of orientation distribution function (ODF). ROI for the initialization of the tractography were selected by overlaying the ODF map on the T2-weighted MRI in order to identify the cisternal trajectory of the cranial nerves with great precision. ROIs were placed on the best identifiable aspect of the cranial nerve in its cisternal segment in the three dimensions: axial, sagittal, and coronal. For each cranial nerve, a single ROI was used; ROIs were cubes and tailored to the anatomical features of each cranial nerve before initiating the tracking. A probabilistic tractography algorithm was used for the tracking of cranial nerves from the ROIs with the following optimized parameters (Jacquesson et al., 2018a): FA cut-off = 0.2–0.3; maximal curvature angle = 45°; minimum fiber length = 10 mm; step size = 0.1 mm. The number of fibers of each nerve to be reconstructed was set from 200 to 1,000 according to the estimated nerve diameter (see Table 1). These tractography results with optimized parameters serve as ground truth for the filtering methods detailed in Section 3.1. In post-treatment, some tractography reconstruction results were filtered manually by an expert in neuroanatomy in order to remove fibers that were away from the anatomy of the nerve.

## 3. Methods

**Figure 1:**
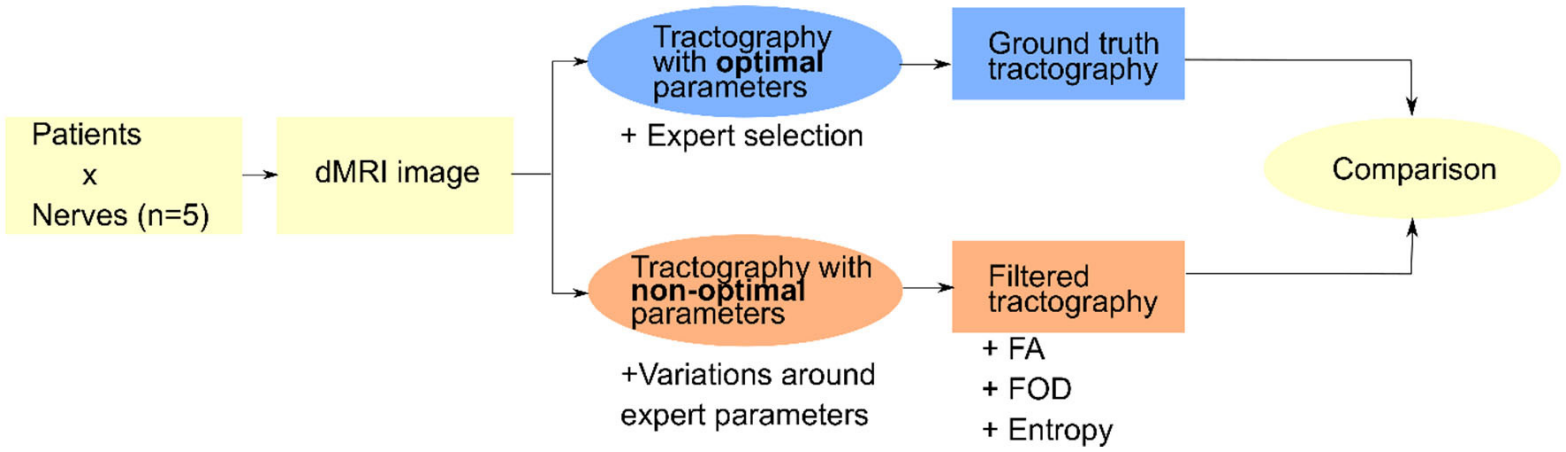
General view of the pipeline used to test different filtering approaches on the basis of non-optimal tractograms with reference to a reference tractogram produced by an expert.

Three filtering methods are tested for their ability to remove erroneous fibers from the tractograms when the tractography parameters are not optimally adjusted. These filtering methods include two state-of-the-art approaches as well as an original approach: they are detailed and explained in section 3.1. Then in order to evaluate the filtering results, an evaluation pipeline is set up, where the filtering results (filtered tractograms) are compared to the reference tractogram as produced with a set of optimal parameters by a neuroanatomy expert. This pipeline is illustrated in Figure 1 and its constituent steps are explained in section 3.2.

### 3.1. Filtering Methods

In this section, we describe the three filtering algorithms used in this study. Before filtering, these methods require the calculation of different parametric maps that are then used to compute—at the scale of a fiber—an indicator of its quality and thus decide whether or not to keep it in the final rendering.

**Figure 2:**
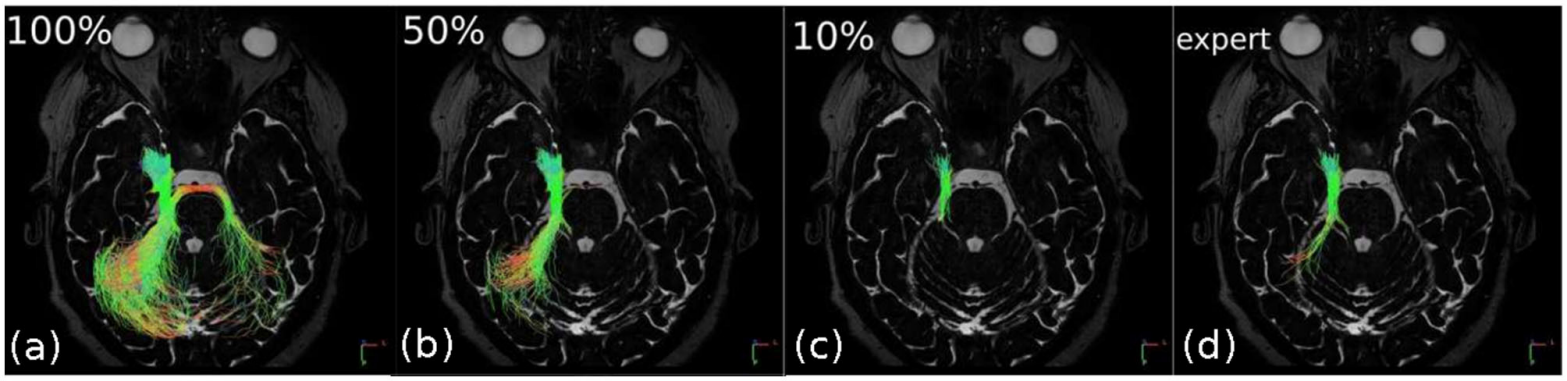
Successive filtering obtained for different percentages of the total number of fibers sorted by decreasing entropy in the right trigeminal nerve (V). The “expert” image shows the result of “ground truth” tractography (for comparison purposes). In **(a)**, 100% of the fibers are depicted and it can be seen that the nerve, due to inaccurate regions of interest (ROI) positioning, has merged to the right with fibers that belong to a different structure of the brain. In **(b,c)**, 50 and 10% of the least entropic fibers are, respectively, displayed; this filtering allows to remove the false continuations and thus to tend toward **(d)** the expected “expert” result.

Figure 2 illustrates filtering results by keeping different percentages of the total number of fibers, where the fiber sorting is performed in an order depending on the filtering method.

#### 3.1.1. Fractional Anisotropy

FA measures the degree of anisotropy of the diffusion of water in a voxel. Without obstacles, water molecules freely diffuse in any direction. This pattern can however be modified by the presence of cell membranes or macromolecules. For example, if the water molecules are confined inside a cell, diffusion only occurs along the main axis of the cell and the diffusion is not isotropic, but anisotropic. The degree of anisotropy can be measured for each voxel of the diffusion MR images from the three eigenvalues (λ_1_, λ_2_, λ_3_) of the diffusion tensor, which describes the diffusion of water using a Gaussian model:


(1) $$\begin{array}{r} FA=\sqrt{\frac{1}{2}}\frac{\sqrt{{\left(\lambda_{1}-\lambda_{2}\right)}^{2}+{\left(\lambda_{2}-\lambda_{3}\right)}^{2}+{\left(\lambda_{3}-\lambda_{1}\right)}^{2}}}{\sqrt{\lambda_{1}^{2}+\lambda_{2}^{2}+\lambda_{3}^{2}}}. \end{array}$$

FA values range from 0 to 1, where 0 represents perfectly isotropic diffusion and 1 represents extremely anisotropic diffusion (Kingsley, 2006). FA values are unitless because they are a ratio of diffusion coefficients. As part of this study, the FA maps were calculated using the function tensor2metric of MRtrix3 software. The mean value of FA along the fibers is computed by trilinear interpolation with the tcksample function of MRtrix3. The fibers are then ranked in the descending order.

#### 3.1.2. Fiber Orientation Distribution

The fiber orientation distribution (FOD) is a spherical probability density function that reveals the orientations and volumes of the underlying fiber bundles. Traditional methods include the estimation of a response function—signal expected in a voxel containing a single bundle of fibers all arranged in a coherent manner—which is then deconvolved from the dMRI signal in order to obtain the FOD (Tournier et al., 2007). The FOD is typically represented as lobes which provide information about the fraction of fibers in the voxel that are aligned along the direction of a lobe. Specifically, the fiber density is correlated with the integral of the FOD lobe and the bundle density to the sum of the segment lengths of fibers (in the voxel) assigned to this lobe. The goal of scale-invariant feature transform (SIFT) approach (Smith et al., 2013) is to assign fibers to the FOD lobes they pass through, so that all fibers contribute to the density of the bundles of the FOD lobes to which they are assigned, depending on their length (Smith et al., 2013) through the voxel of interest as well as a variable contributing weight (Smith et al., 2015):


(2) $$\begin{array}{r} \mu =\underset{s:|s_{l}|>0}{\sum }|s_{l}|.e_{s}^{F}, \end{array}$$

where each fiber *s* that traverses the lobe *l* contributes the bundle density according to the product of its length |*s*_*l*_| through the voxel, and contributing weight $e_{s}^{F}$.

The underlying idea is to determine a vector of contributing weights *F*, such that when the contribution of each fiber is weighted accordingly in this vector, the bundle densities match the FOD lobe integrals throughout the all image. The FOD map is computed from the diffusion MR images using MRtrix3 dwi2fod function. On the basis of SIFT method, the associated *F* map is associated with the fibers by the tcksift2 function of MRtrix3. The fibers are then ranked in the ascending order.

#### 3.1.3. Entropy

**Figure 3:**
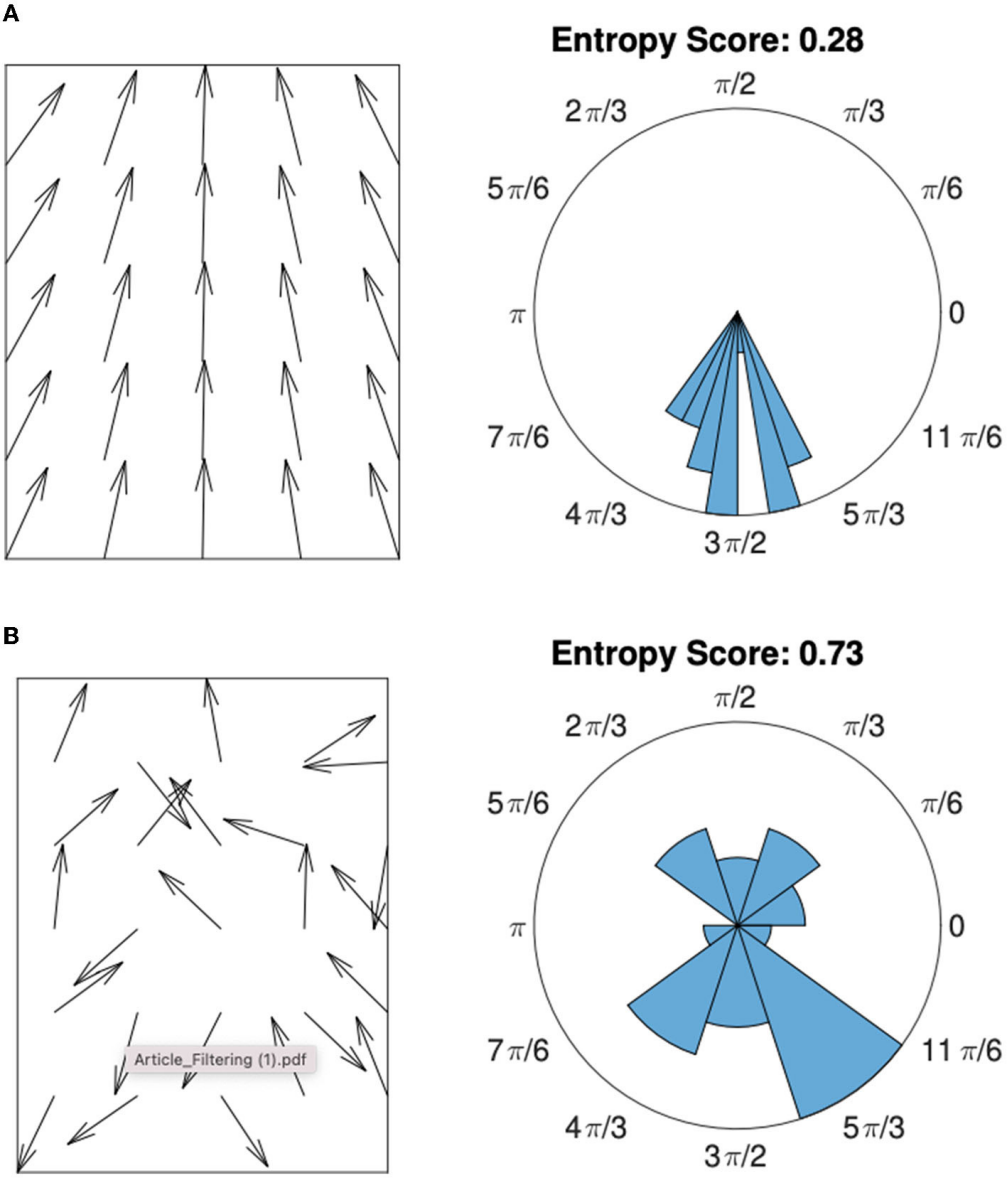
Orientation histogram associated with vector fields of low entropy **(A)** and high entropy **(B)**. The score given in the right column corresponds to the entropy value of the central vector calculated using a 3 × 3 neighborhood and a circular orientation histogram with 10 bins. The more the vector field is scattered, the higher the entropy value.

We propose a third way of filtering on fibers which is based on entropy and is an original contribution proposed within the framework of this work. Shannon's entropy is used here to measure the homogeneity of the orientation of tractography fibers. The entropy of a vector field is defined by the entropy of the histogram of the vector orientations as follows:


(3) $$\begin{array}{r} e\left(X\right)=-\underset{i=1\ldots n}{\sum }p\left(x_{i}\right)log_{2}\left(p\left(x_{i}\right)\right), \end{array}$$

where *x*_*i*_ is the bin *i* of the histogram of vector orientations and *p*(*x*_*i*_) the probability for a vector to be in the bin *x*_*i*_. The probability *p*(*x*_*i*_) is calculated as:


(4) $$\begin{array}{r} p\left(x_{i}\right)=\frac{C\left(x_{i}\right)}{\sum_{i=1\ldots n}C\left(x_{i}\right)}, \end{array}$$

with *C*(*xi*) the number of vectors in the bin *x*_*i*_. Figure 3 illustrates this process in a two-dimensional case for a case of orientation disorder (Figure 3A: high entropy) and orientation coherence (Figure 3B: low entropy).

To compute the local entropy of tractograms, the fibers are first converted to a vector field encoding their 3D local orientations. This vector field could be obtained from the main eigenvector of the diffusion tensor estimated from the raw dMRI data. However, this method is very sensitive to noise, particularly because of the low resolution of the dMRI data (2 mm) compared to the diameter of the nerves (2–10 mm: see Table 1). We therefore propose to reconstruct a vector field from the fibers themselves. Considering that fibers are sampled more than 10 times finer than the dMRI voxel, the resolution of the final vector field can be drastically improved. The fibers are transformed into a 3D image of local fiber density information, and a map of the maximum intensity gradient direction of this image is calculated using a 3 × 3 × 3 neighborhood according to the method in Xu and Prince (1998). Since the gradient orientation is normal to the actual fiber orientation, the vectors are reoriented according to the average of the vector products of the central voxel and its neighbors in the 3 × 3 × 3 neighborhood.

Then, each voxel of coordinates (*x, y, z*) in the vector field is associated to a small cubic neighborhood *n* × *n* × *n*. Considering 3D vectors, a spherical orientation histogram in the neighborhood of the considered voxel is computed. This is achieved in 3D by decomposing the unit sphere into patches of equal area (Leopardi, 2006), and using the cones connecting the patches to the center of the sphere as bins for the orientation histogram. The entropy is computed from the spherical orientation histogram as in Equation (3) and the entropy value is assigned to the corresponding voxel (*x, y, z*) in the 3D entropy map *E*(*x, y, z*).

The entropy map depends on two parameters; the number of bins *n* used in the histogram and the size of the neighborhood considered to build the histogram. In our case, the parameters are chosen by taking into account priors on the dMRI acquisition and the anatomy of the cranial nerves. The number of bins corresponds roughly to the number of diffusion directions used in dMRI acquisition (*n* = 32) and the neighborhood size is proportional to the diameter of the considered nerve, as given in Table 1.

The coordinates of the fibers are re-written in the referential of the 3D vector field used to compute the entropy. Each fiber is weighted by a score α. This score is computed from the entropy map *E* as follows:


(5) $$\begin{array}{r} \alpha =\frac{1}{\left|X\right|}\underset{\left(x,y,z\right)\in X}{\sum }E\left(x,y,z\right), \end{array}$$

where *X* is the set of coordinates of the considered fiber.

### 3.2. Evaluation

#### 3.2.1. Non-optimized Tractography Dataset

The performance of the proposed filtering methods is evaluated in a context of non-optimized tractography (i.e., non-expert operators). For this, a non-optimized database of cranial nerve tracking was generated. The ROI location and size and the FA threshold were identified as parameters of interest for this study because their optimal value strongly depends on the acquisition, pathology, and anatomical variability of the patient (Jacquesson et al., 2018b). They are likely to have a strong impact on the tractography results.

For each of the cranial nerves considered in this study, the fiber tacking is performed with non-optimal tractography parameters chosen as described in Table 2. The range of FA threshold values was selected with regard to recent state of the art (Jacquesson et al., 2018b) and the size and location of the ROI on the basis of inter-operator variability observed in previous work (Colon-Perez et al., 2016). It should be noted that FA threshold is only decreased and ROI size only increased because a high value of FA threshold or a very small size of ROI tend to truncate the nerve and therefore loose information, a case in which a filtering algorithm has little interest. The ROI size and position were impaired by applying dilation and translations to the optimal ROI designed by the expert. Such changes are done proportionally to the diameter *D* of the studied nerve (see Table 1) to have a comparable impact on each nerve. The influence of each tractography parameter is independently studied so that the other parameters are set to their default optimal values given in section 2.4.

**Table 2:** Values used for each tractography parameter in order to build the “non-optimal” database.

**Parameter**	**Dataset 1**	**Dataset 2**	**Dataset 3**	**Dataset 4**	**Dataset 5**
FA threshold	oFA	oFA-0.03	oFA-0.06	oFA-0.1	–
ROI size	oROI_*s*_	oROI_*s*_+*D*/5	oROI_*s*_+2*D*/5	oROI_*s*_+3*D*/5	oROI_*s*_+4*D*/5
ROI vertical	oROI_*l*_-2*D*/5	oROI_*l*_-*D*/5	oROI_*l*_	oROI_*l*_+*D*/5	oROI_*l*_+2*D*/5
translation					
ROI horizontal	oROI_*l*_-2*D*/5	oROI_*l*_-*D*/5	oROI_*l*_	oROI_*l*_+*D*/5	oROI_*l*_+2*D*/5
translation					

*D is the diameter of the nerve, as given in Table 1. oFA, oROI_s_, and oROI_l_ stand for, respectively, optimal values of FA, regions of interest (ROI) size, and location*.

Because we drift away from optimal parameters, in some cases only few or no fibers could be tracked by tractography. Filtering being is useless in such cases, they were excluded from our validation study.

#### 3.2.2. Quality Metrics

**Figure 4:**
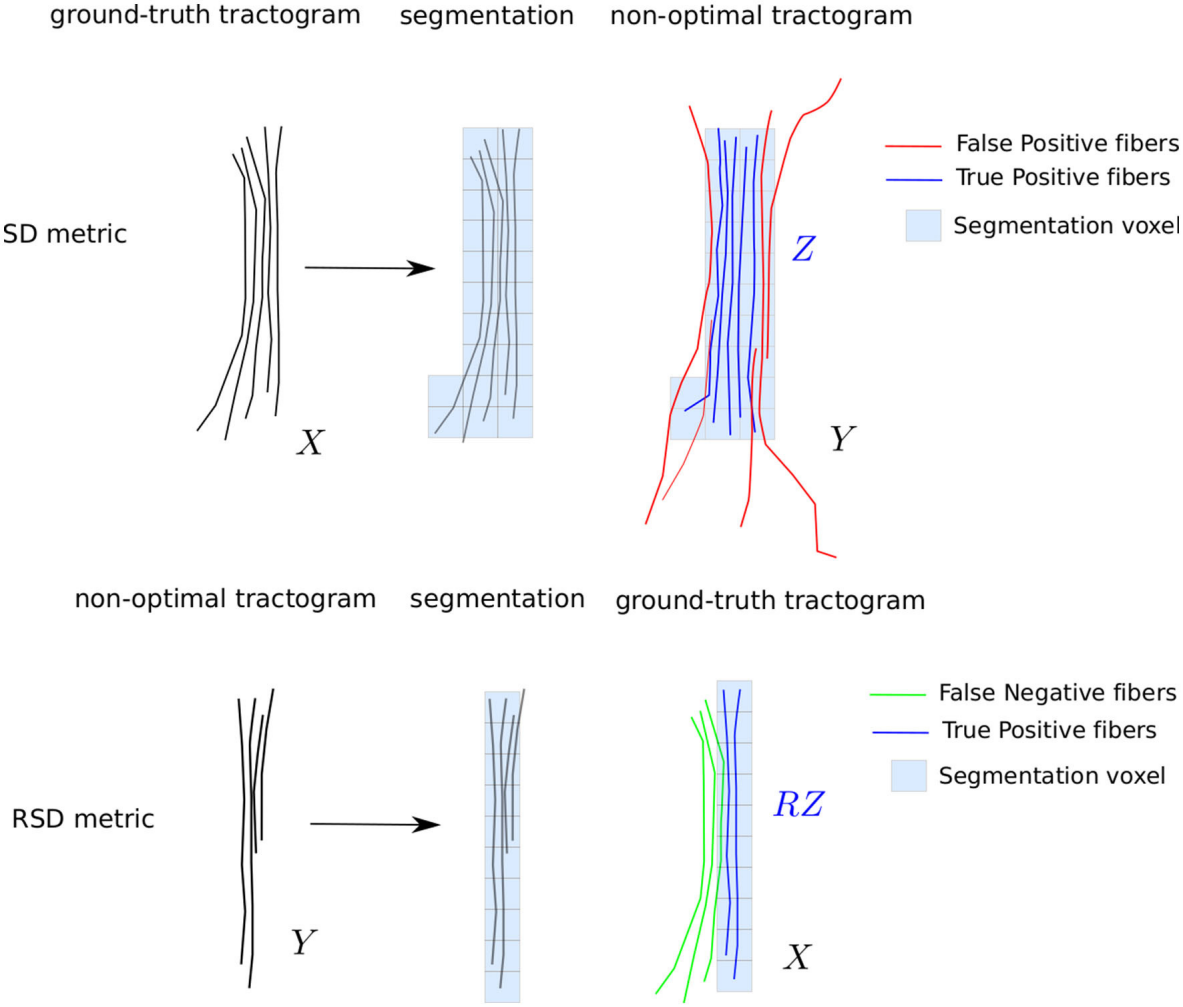
Illustration of the quality metrics *SD* and *RSD* used to evaluate the sensibility to tractography parameters and the filtering performance.

The stochastic aspect of the probabilistic tractography algorithm makes it difficult to compare two tractograms. To allow a quantitative comparison, the performance of the filtering methods is assessed with a metric derived from the Sørensen-Dice score (Dice, 1945). The Sørensen-Dice score measures the similarity of two sets of elements as the ratio between the number of elements in their intersection and the sum of the elements in both sets. It ranges from 0 (the ensembles are disjoint) to 1 (the ensembles are overlapped). In this work, it is used to compare two tractograms; the ground truth tractogram *X* and the non-optimized tractogram *Y*. Due to the stochastic and continuous nature of tractography fibers, an original way to define their intersection, named *Z*, is proposed and illustrated in Figure 4 on the top. The ground truth tractogram is first discretized on the basis of the original medical image voxels to produce a segmentation of the nerve of interest. The fibers of the non-optimized tractogram *Y*, which are included in the ground truth segmentation, forms the intersection set *Z*. We define our Sørensen-Dice score as :


(6) $$\begin{array}{r} SD=\frac{2|Z|}{\left|X|+|Y\right|}. \end{array}$$

where *X* is ground truth tractogram and *Y* is one of the non-optimal tractograms (see Table 2), and *Z* is their intersection as defined above. The cardinality operator || refers to the number of fibers in the tractograms.

This metric is penalizing as it takes into account not only the position of the fibers but also their density. The maximum score can be obtained only if the number of fibers in the intersection and the number of fibers in the ground truth tractogram are the same. A tractogram containing only true positive fibers but with a lower number than in the ground truth is penalized. This is a welcomed feature in the design of our quality metric, as a too low fiber density may impair visualization of the nerve. This metric gives a lot of weight to false positive (i.e., spurious) fibers, which makes it is appropriate to measure the contribution of the filtering methods. However, it is not efficient for the detection of false negative fibers (i.e., missing nervous fibers), which cause incomplete or truncated nerves to obtain a high scores.

To take into account the truncature effect, we proposed another metric, that we call reverse Sørensen-Dice score *RSD*. It differs from the *SD* score in the way the intersection between *X* and *Y* is computed. In *RSD*, the non-optimized tractogram *Y* is segmented instead of *X*. The intersection, called *RZ*, is composed of the fibers of the ground truth *X* which are included in the segmentation of *Y* (see Figure 4). The *RSD* metric can be written as:


(7) $$\begin{array}{r} RSD=\frac{2|RZ|}{\left|X|+|Y\right|}. \end{array}$$

This metric penalizes the false negative fibers; a truncated nerve will obtain a low score. In the next paragraph, we describe the specific metrics derived from *SD* and *RSD* in order to investigate (1) the sensitivity of tractogram to non-optimal tractography parameters and (2) the contribution of the filtering algorithms to improve the quality of the tractograms.

### 3.3. Sensitivity to Tractography Parameters

The study of the sensitivity to tractography parameter requires to evaluate both the unwanted fibers (false positive) and the missing fibers (false negative). In this case, both *RSD* and *SD* scores provide relevant information. This study does not investigate the filtering methods; we aim at comparing the non-optimized tractograms without any filtering, as described in section 3.2.1, to the ground truth. For this, two metrics are used. *SD*_init_ (respectively, *RSD*_init_) refers to the *SD* score (respectively, *RSD* score) between the ground truth tractogram and non-optimized tractograms before filtering.

### 3.4. Filtering Performance

To evaluate the performance of the filtering methods, we will study two metrics based on the *SD* score. The maximum *SD* score between the ground truth tractogram and the non-optimized tractograms after filtering is noted *SD*_max_. The score *SD*_max_ is the maximum of *SD* scores for filtering percentages that vary between 0 and 100% with a step of 1%. It represents the ability of the filtering algorithm to reach the expert result.

The performance of a filtering method is assessed by computing the difference *SD*_diff_ = *SD*_max_−*SD*_init_. *SD*_diff_ reflects the gain brought to the tractogram by the filtering method. If the initial tractogram is already of good quality, the gain will be low, whereas if the initial tractogram is noisy or erroneous, the gain might be more important. The most efficient filtering methods will achieve the highest gain.

These two indices, *SD*_max_ and *SD*_diff_, will be compared between nerves, tractography parameters, and filtering methods on the same data samples. The statistical test used is the paired Wilcoxon signed-rank test, since non-normality was observed for these data and samples are not independent.

## 4. Results

In this section, we present the different results obtained. First, we observe the sensitivity of the tractograms with regard to the nerves and the variations applied to tractography parameters as given in Table 2. Then, we investigate the contribution of the filtering approaches from a general point of view and with regard to each tractography parameter variation. Finally, we study the optimal filtering threshold for each of the filtering methods for the sake of automation.

### 4.1. Sensitivity

Before looking at the performance of the filtering, we study the sensitivity of the tractograms to the variation of each tractography parameter with respect to the different nerves. This sensitivity is expressed by the *SD*_init_ index introduced in section 3.4.

#### 4.1.1. Sensitivity to Nerves

**Figure 5:**
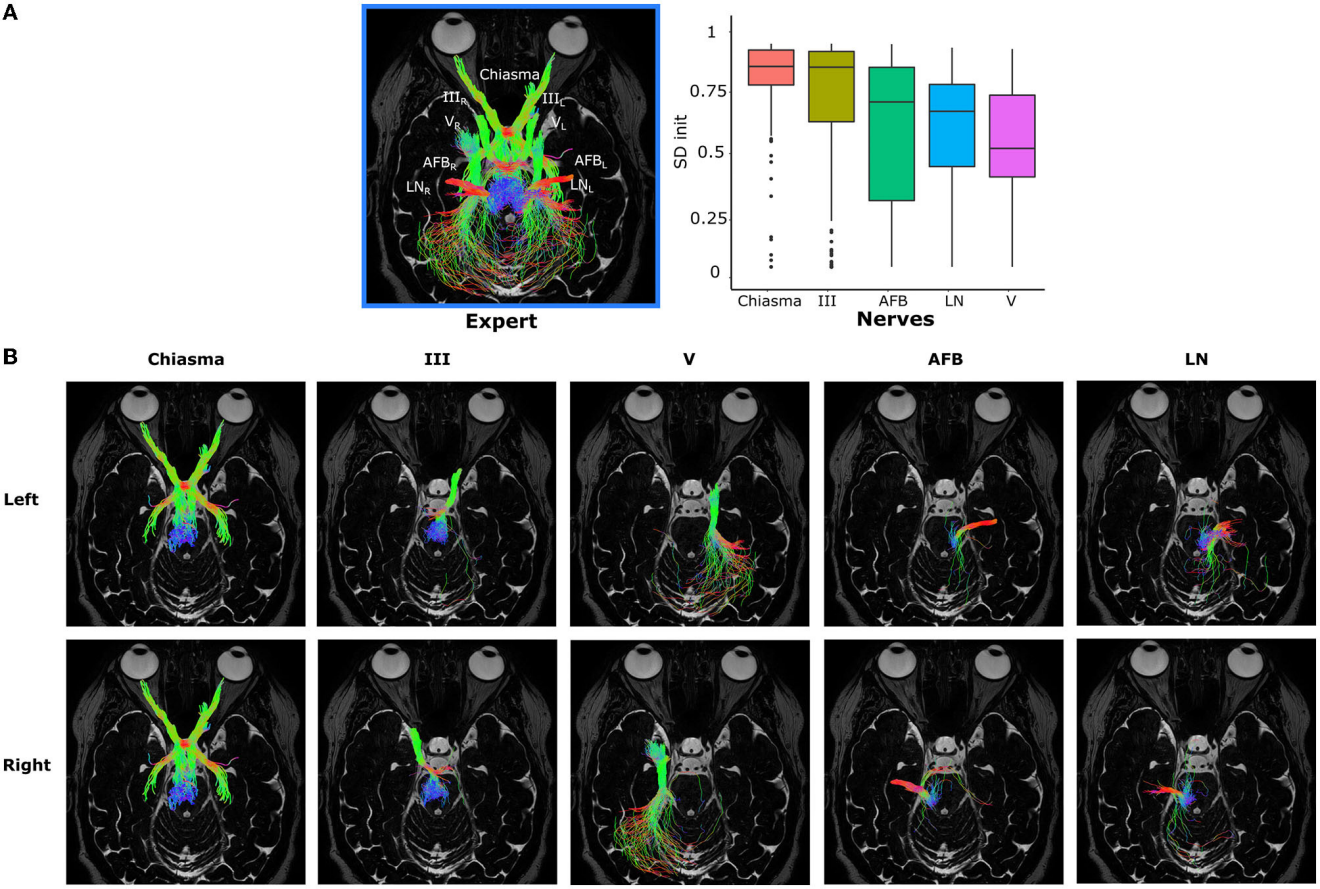
**(A)** On the left, the five nerves of interest represented for a given patient and the two hemispheres (left L and right R). On the right, the distribution of *SD*_init_ according to the five nerves of interest for all patients and all the variations imposed around the values of optimal tractography parameters. **(B)** Independent representation of each nerve. The different nerves are represented in column according to the following order: Chiasma, III, V, AFB, LN, and in line according to the left then right hemisphere.

Figure 5A shows on the left the different nerves in a patient for the left and right hemisphere. The disparity in the values of *SD*_init_ for the different nerves can be observed in Figure 5A on the right: this boxplot represents the distribution of *SD*_init_ for each of the nerves on the basis of all the disturbances created according to the non-optimal database detailed in section 3.2.1. Table 3 gives the *p*-values associated with the Wilcoxon tests carried out two by two for each pair of nerves.

**Table 3:** *P*-values from paired Wilcoxon signed-rank tests comparing two by two each nerve based on *SD*_init_ index.

	**Chiasma**	**III**	**AFB**	**LN**
III	0.18419			
AFB	6.4e-14	1.1e-12		
LN	<2e-16	<2e-16	0.17866	
V	<2e-16	<2e-16	0.00021	0.00229

The nerves form two groups: one made up of Chiasma and III and another of V, LN, and AFB. The nerves Chiasma and III have roughly the same initial quality of tractography with a median of, respectively, *SD*_init_ = 0.898 and *SD*_init_ = 0.895 and are not significantly different (see Table 3). The median values for AFB (*SD*_init_ = 0.739), LN (*SD*_init_ = 0.697), and V (*SD*_init_ = 0.531) are significantly lower to Chiasma and III. Within the group made of AFB, LN, and V nerves, LN and AFB are not statistically different (see Table 3); V is statistically different from LN and AFB but with *p*-values much lower than those obtained for the group of Chiasma and III nerves.

All this tends to say that the initial quality of the tractograms is not equal between the nerves: Chiasma and III are less sensitive to parameter variation than AFB, LN, and V. This fact can be explained by the localization of the nerves: in fact, AFB, LN, and V are closer to the back of the skull (as depicted in Figure 5B) and therefore to other nervous structures which can, if the tractography parameterization is not optimal, be reconstructed with the nerve of interest.

#### 4.1.2. Sensitivity to Tractography Parameters

**Figure 6:**
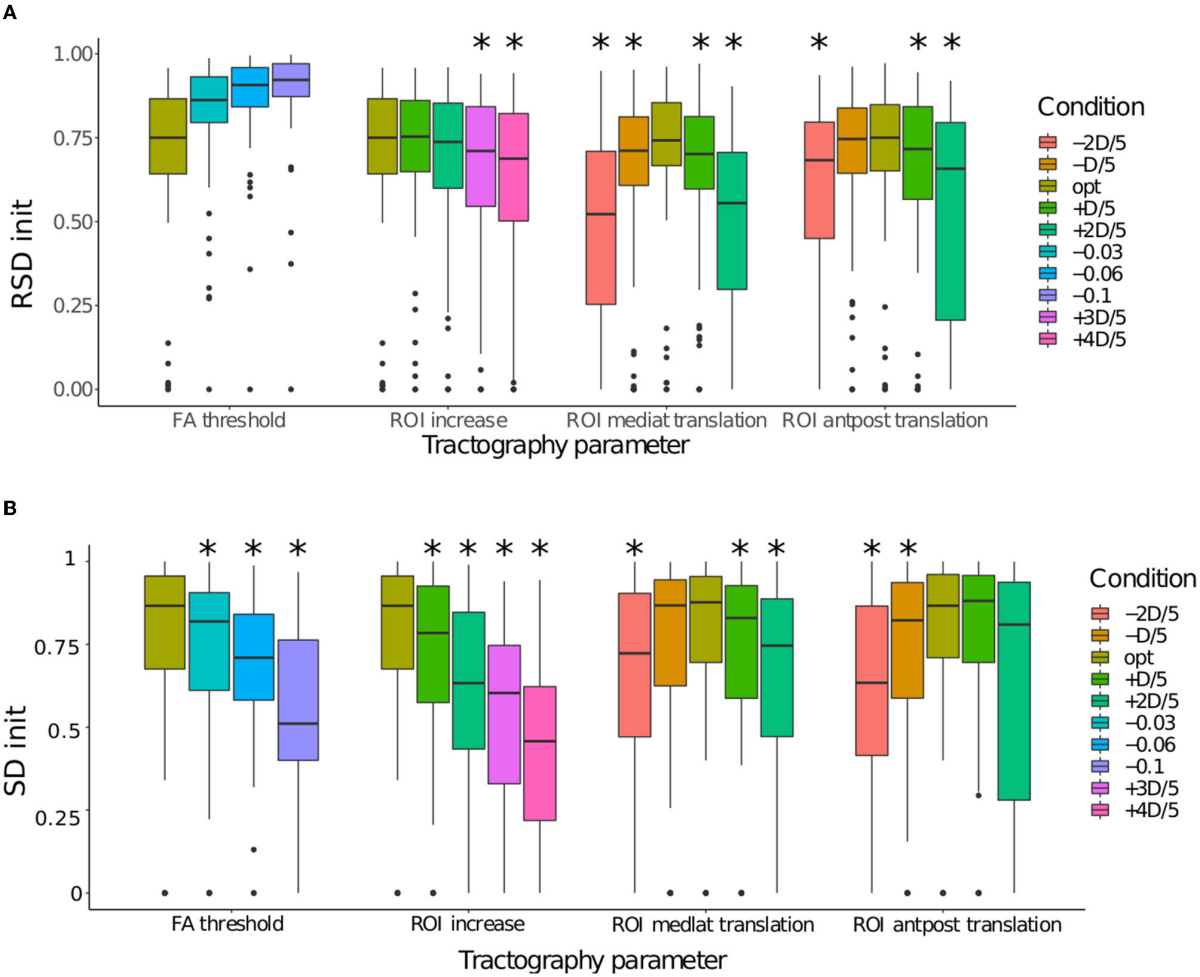
**(A)** Comparison of the distributions of *RSD*_init_ according to each condition for the four tractography parameters: (1) FA threshold, (2) ROI size, (3) ROI mediolateral translation, and (4) ROI anteroposterior translation. **(B)** Comparison of the distributions of *SD*_init_ according to each condition for the four tractography parameters: (1) FA threshold, (2) ROI size, (3) ROI mediolateral translation, and (4) ROI anteroposterior translation. When a condition is significantly smaller to the optimal condition, it is marked with *.

The impact of the tractography parameters on the values of *RSD*_init_ and *SD*_init_ can be observed in Figure 6. In order to study the impact of each parameter variation more in detail, each parameter was varied independently, while the others are left at their optimal value. Figure 6 represents the effect of the variation of each tractography parameter on the initial quality of the tractograms for all nerves together.

The *RSD*_init_ score are given in the panel A of Figure 6, allowing to evaluate whether a change in parameters causes truncature of the nerve (low scores) or the fibers tracked covers the whole nerve (high scores). As it was expected, the decrease of the FA values causes the *RSD*_init_ to increase, as more spurious fibers are produced. The increasing of the ROI size also preserves the shape of the nerve, although the *RSD*_init_ score becomes significantly lower when the increase factor exceeds +3*D*/5. However, the ROI translation significantly lowers the *RSD*_init_. Two types of displacement (mediolateral and anteroposterior translations) were modeled with two directions of translation with regard to the optimal one. This explains a non-monotonic evolution for these two ROI localization parameters. This drop was expected: as the ROI is moved away from its optimal position, fibers outside of the nerves are tracked, causing the nerve to appear incomplete. In such a case, the filtering algorithm might not be efficient as it can only remove the spurious fibers and not add the missing ones.

The *SD*_init_ score are given in the panel B of Figure 6, allowing to evaluate whether a change in parameters causes the generation of spurious fibers to be removed (low scores). It appears that for FA threshold the *SD*_init_ is already significantly lower for the tractograms with the smallest variation (FA-0.03). Same pattern of evolution is observed for the ROI size, with a slightly larger drop. In both cases, a lot of extra fibers are produced, hindering the visualization. In comparison, the variations observed for the position of the ROI seem smaller. For ROI with a mediolateral translation, the difference between all the tractograms is immediately significant for a lateral move (toward the right on the boxplot), but only for the value oROI_*s*_-2*D*/5 for a move in the medial direction. For ROI with an anteroposterior translation, only the forward displacement is already significant from the value oROI_*s*_-*D*/5. It should be noted that the *RSD*_init_ and *SD*_init_ scores of the optimal parameters is not at 1 because it includes the variability due to the probabilistic nature of the tractography algorithm used in this study (see section 2.4). In conclusion, lowering the FA parameter or increasing the ROI size results in the production of a lot of erroneous fibers while the ROI translation causes both a truncature of the nerve and erroneous fibers.

### 4.2. Filtering Contribution

In this section, we evaluate the ability of the three filtering methods described in section 3.1 to remove the erroneous fibers and thereby improve the non-optimized tractograms. This performance is expressed by the *SD*_diff_ index introduced in section 3.4.

#### 4.2.1. General Performances

Regarding the overall performance of the three filtering methods evaluated using the *SD*_*mathrmdiff*_ index and on the basis of all the tractograms of all nerves and patients, the median is equal to 0.049 for entropy, 0.018 for FA, and 0.067 for FOD. It therefore appears that the gain in filtering on the basis of the same data is in favor of the FOD and entropy methods.

**Figure 7:**
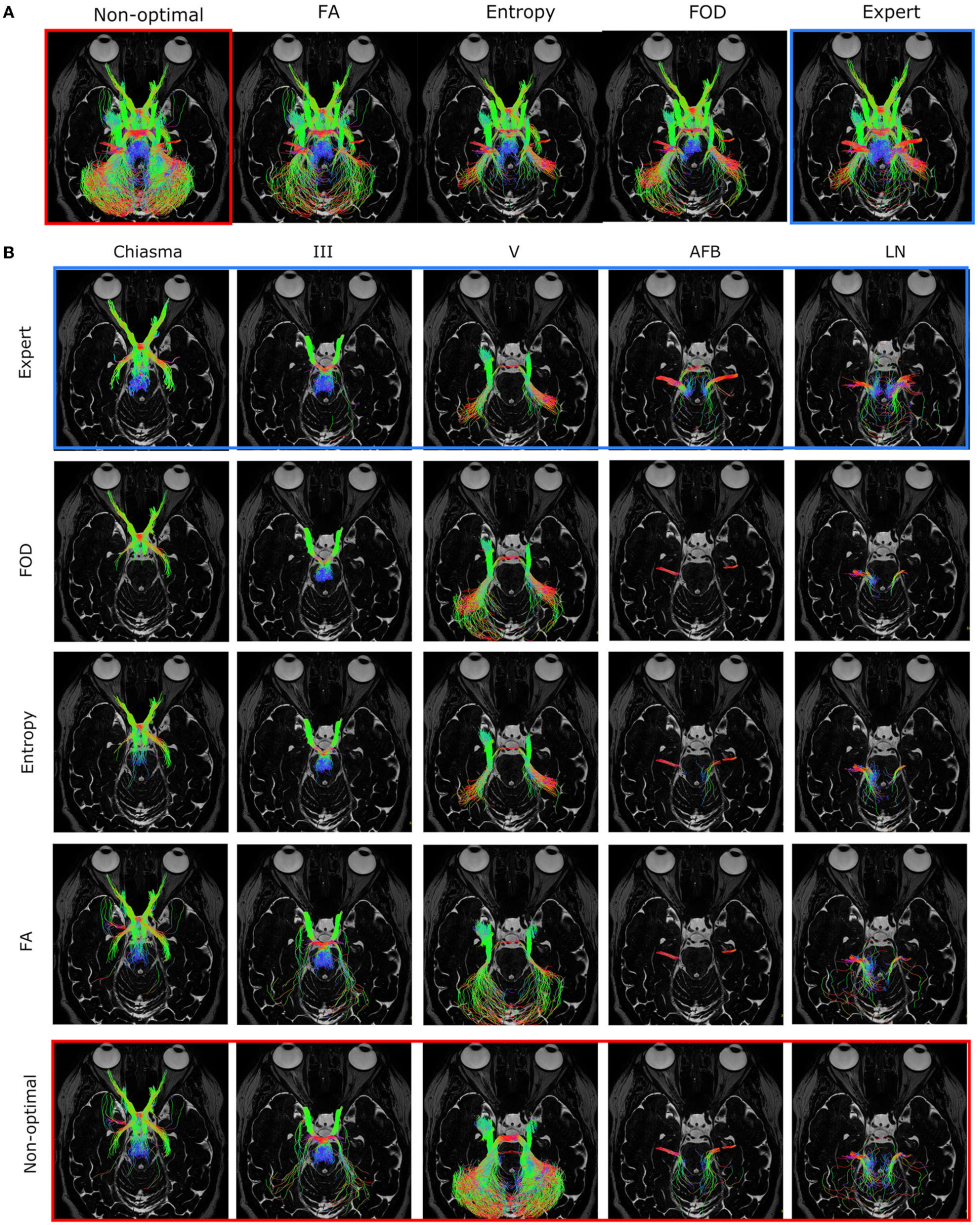
**(A)** Optimal filtering obtained for the three different filtering methods [FA, entropy, and fiber orientation distribution (FOD), respectively] for all cranial nerves of a specific patient in the case of a regions of interest (ROI) that is too large. **(B)** Independent representation of each nerve. The different nerves are represented in column according to the following order: Chiasma, III, V, AFB, LN, and in line according to the different filtering methods. The “expert” image shows the result of the “ground truth” tractogram and was used for the choice of the optimal threshold. The “non-optimal” image shows a noisy tractogram obtained with poor initialization of the tractography parameters.

Figure 7 represents the overall performance of the three filtering methods qualitatively for a patient and for all nerves combined. On the basis of visual observations made from Figure 7A, the quantitative results obtained are confirmed: the entropy and FOD methods give results closest to the ground truth and relatively equivalent quality, while the FA method produces a visually different filtering result which is less in agreement with the ground truth, in particular for the nerves identified in section 4.1 as the most variable, namely V, LN, and AFB. Figure 7B allows to study in more detail, nerve by nerve, the effect of the filtering. First observation, the nerves V and III are the most impacted by the increasing of ROI size and are therefore those which benefit the most from the filtering with the best results for the FOD and entropy methods, which make it possible to approach the ground truth more closely.

#### 4.2.2. Tractography Parameters Influence

**Figure 8:**
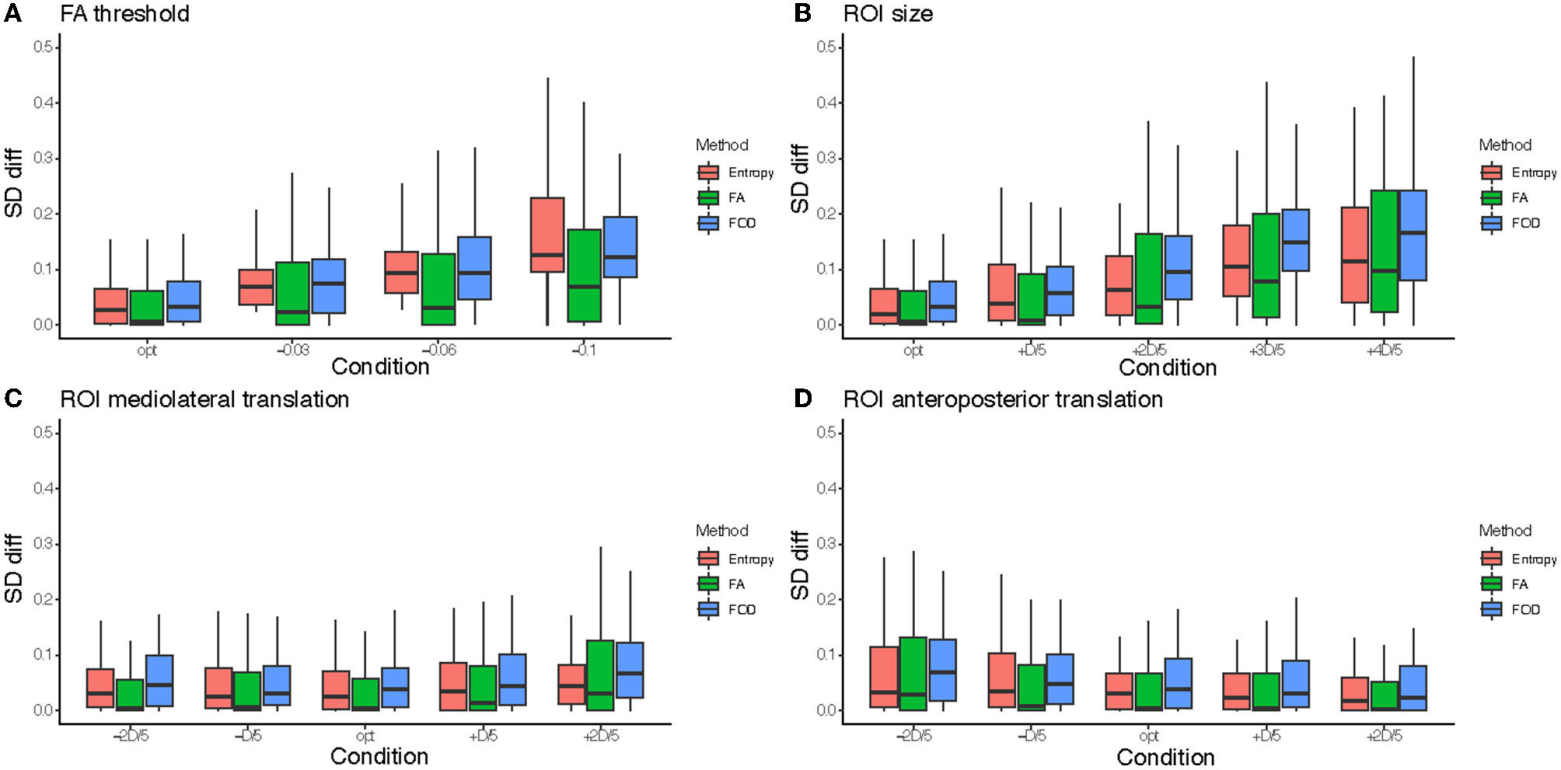
Comparison of the distributions of *SD*_diff_ according to each condition for the four tractography parameters: **(A)** FA threshold, **(B)** regions of interest (ROI) size, **(C)** ROI mediolateral translation, and **(D)** ROI anteroposterior translation. For each parameter, *SD*_diff_ is displayed according to each condition and for all three methods [entropy, FA, and fiber orientation distribution (FOD)].

Figure 8 shows the performance of the three filtering methods according to the different tractography parameters based on the *SD*_diff_ index. In general, it can be observed that the further we move away from the optimal value of the parameter, the greater the filtering gain is, independently of the filtering method. This can be explained by the fact that tractograms produced with non-optimal parameters are more degraded. Their *SD*_init_ index is lower, which leaves more room for improvement by the filtering methods. If we compare the filtering methods according to the four tractography parameters, we can observe that FOD provides the best results in the ROI size increase case (significantly different from FA with a *p* <4.9e-14 and entropy with a *p* < 1.7e-08) and better results but very close to entropy for the parameters related to the location of the ROI (significantly different from FA with a *p* < 1.8e-11 and entropy with a *p* < 0.0478). Concerning the parameter on the FA threshold, the entropy method is competitive or better. The FA method offers lower gains for the four tractography parameters, as observed in the overall performances.

**Figure 9:**
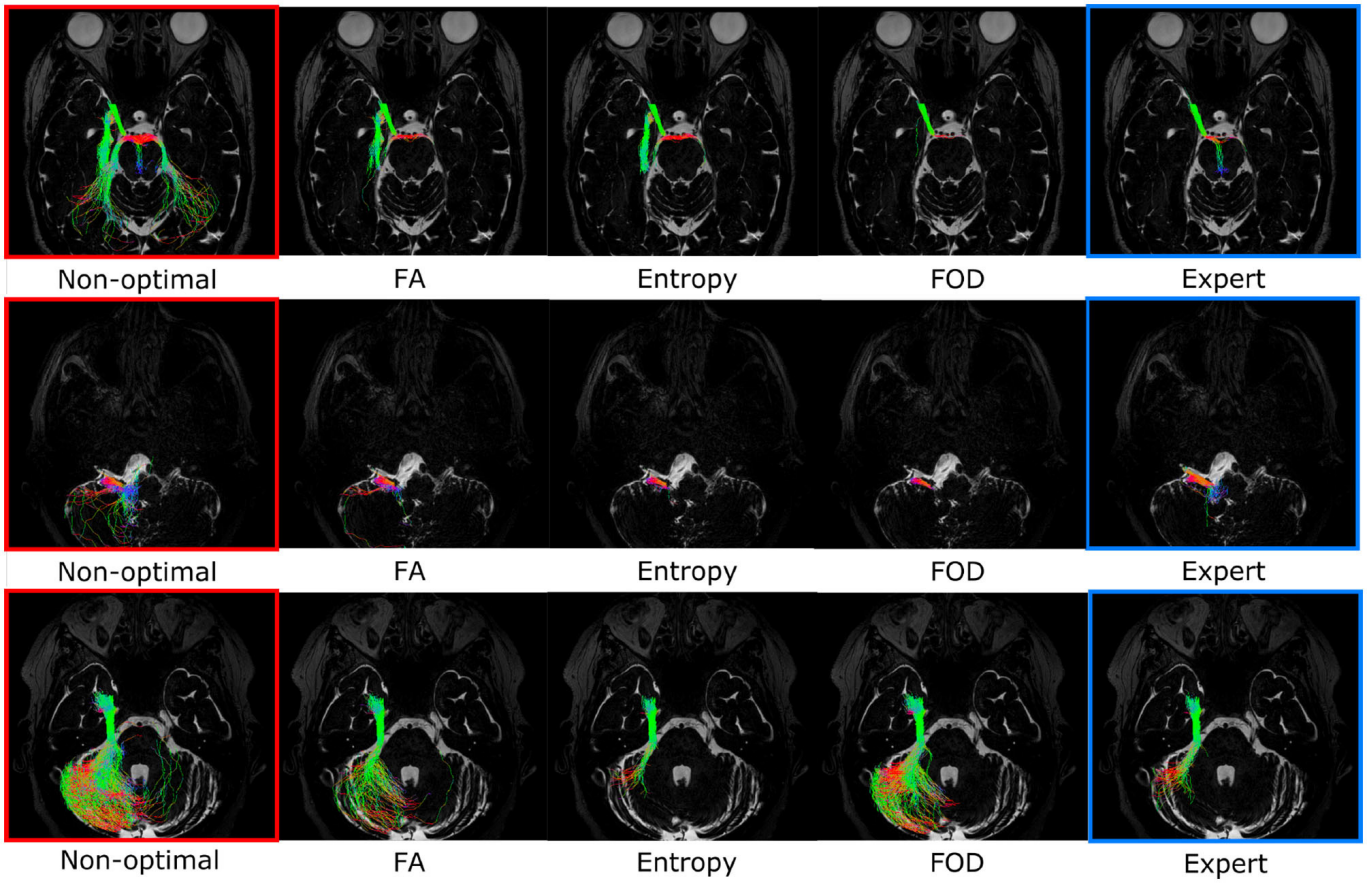
Optimal filtering obtained for the different filtering methods (FA, entropy, and fiber orientation distribution [FOD], respectively) for specific cranial nerves and patients. The “expert” image shows the result of the ground truth tractogram. The “non-optimal” image shows the noisy tractogram obtained with poor initialization of the tractography parameters. **First line**: Illustration of the filtering on the right occulomotor nerve (III) in the case of regions of interest (ROI) that is too large. This case is more efficiently treated by the FOD-based method. **Second line**: Optimal filtering obtained for the right fascial and cochleo-vestibular nerves group (LN) in the case of poor placement of the ROI. This case is more efficiently treated by methods based on FOD and entropy. **Third line**: Optimal filtering obtained for the right trigeminal nerve (V) in the case of non-optimal FA threshold. This case is more efficiently treated by entropy-based method.

Figure 9 gives a qualitative representation of the performance of the three filtering methods according to the different tractography parameters for different patients and nerves. The visual results presented in Figure 9 confirm the quantitative results obtained from the analysis of Figure 8: the FOD method provides the best results in the case of a too large ROI (case of line 1), concerning the localization of the ROI the entropy and FOD methods are competitive (case of line 2), finally concerning the FA threshold the method based on the entropy provides the results closest to the ground truth.

**Figure 10:**
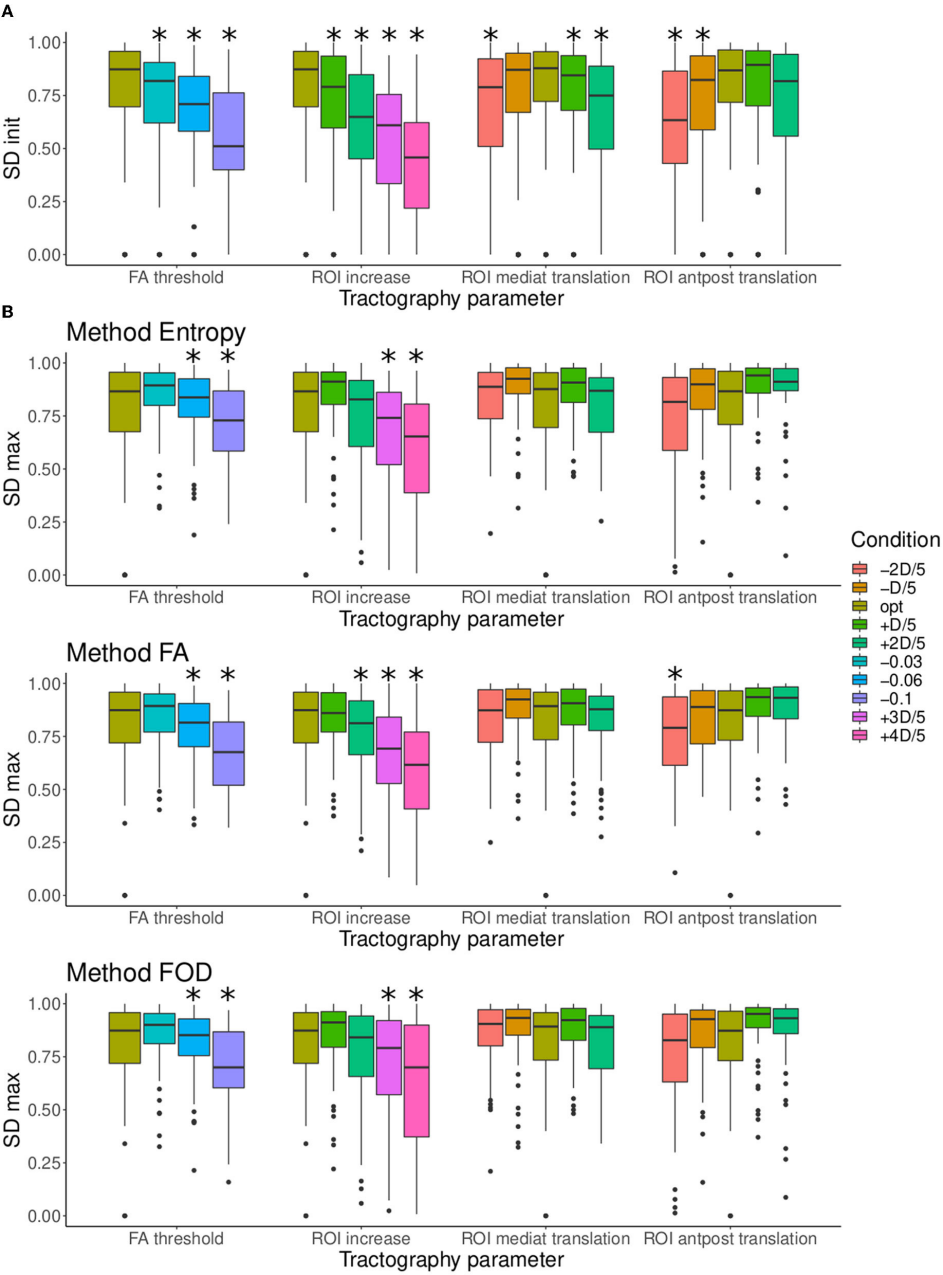
**(A)** Comparison of the distributions of *SD*_init_ according to each condition for the four tractography parameters FA threshold, regions of interest (ROI) size, ROI mediolateral translation, and ROI anteroposterior translation. **(B)** Comparison of the distributions of *SD*_max_ (after filtering) according to each condition for the four tractography parameters and to the three filtering methods (entropy, FA, and FOD), each represented in a different row. It should be noted that in **(B)** for optimal conditions *SD*_init_ is given instead of *SD*_max_ because the optimal tractograms are not filtered. When a condition is significantly smaller to the optimal condition, it is marked with “*”.

To analyze more generally the contribution of filtering, we wanted to compare Figure 6 before filtering with the results after filtering for the three tested methods. Figure 10 shows whether the difference between the optimal condition and the degraded conditions of Table 2 are still considered significant after filtering. It turns out that for the FA threshold and ROI size parameters, only the tractogram obtained with the parameter condition the furthest from the optimal remain significantly different (oFA-0.06, oFA-0.1 and oROI+3D/5, oROI+4D/5). In the case of the displacement of the ROI, for the entropy and FOD methods, none of the parameter variations trigger a significantly lower tractogram quality. The FA method is less efficient in this case. In conclusion, the filtering effectively makes it possible to move away from the optimal parameters while guaranteeing to find by post-processing tractograms close to those of the ground truth.

#### 4.2.3. Optimal Filtering Threshold

**Figure 11:**
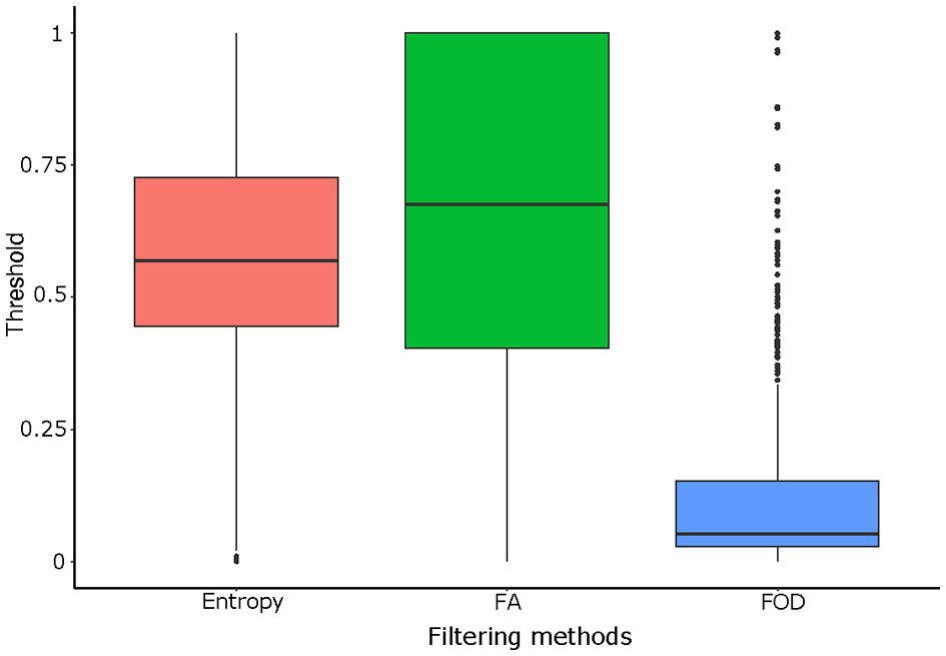
Comparison of the distributions of the optimal filtering threshold according to each filtering method [entropy, FA, and fiber orientation distribution (FOD)].

Figure 11 shows, for each filtering method, the distribution of the optimal filtering thresholds in order to reproduce the ground truth as closely as possible (index *SD*_max_ introduced in section 3.4). These thresholds were normalized by patient with regard to the minimum and maximum indices of entropy, FA, and FOD maps, respectively. Figure 11, therefore, represents the variability of the adjustment of the optimal threshold for the different patients and the possibility of its automation. For the entropy, the median value is equal to 0.569, for FA to 0.676, and for FOD to 0.052. The distribution of the thresholds for the entropy method is evenly distributed around the median, while the interquartile distribution for the FA method is more spread above the median. The FOD distribution has a lower median than the other methods and has many values identified as outliers. These two points can make automation difficult for the FOD method.

## 5. Discussion

In this study, we studied the impact of variations in the tractography parameters on the quality of the tractograms, with regard to the ground truth produced by an expert. The contribution of three filtering methods to correct these perturbations, including a new algorithm, was evaluated with several levels of observation. We have found that filtering increases the overlap with the tractograms produced by the expert, compensating efficiently for a poor tractography parameter optimization. The FOD method seems to be the most efficient filtering method, followed by the new entropy-based method we proposed, which benefits from an optimal threshold that is easier to select. These encouraging results pave the way for the automation of the tractography process.

### 5.1. Cranial Nerves and Tractography

Cranial nerves are particularly difficult to track because of their small sizes (2–10 mm, see Table 1) and their proximity to other structures, especially the brainstem, which can cause false continuations for the tracing of a specific nerve (Yoshino et al., 2016; Jacquesson et al., 2019). According to our study on the sensitivity of the tractograms on the basis of poorly optimized tractography parameters, the most sensitive nerves would be V, LN, and FN. This is in line with results from the literature, which show that the smaller structures tend to be more difficult to follow in tractography (Hodaie et al., 2010; Garcia-Fidalgo and Ortiz, 2013; Jeurissen et al., 2019).

Due to their anatomical characteristics, the cranial nerves are all the more difficult to follow when the tractography parameters are poorly optimized. In this context, it could be observed that it is the size of the ROI which seems the most critical, then the threshold of FA and finally the location of the ROI. As the ROI are drawn in the cisternal segment in the three dimensions, a slight displacement (as achieved in our study) results in a more limited distortion on the resulting tractogram. It should be noted that these significant distortions correspond to parameters that could be selected for the cranial nerves tractography—even by experts—with regard to the state of the art (Jacquesson et al., 2018b). More specifically, they can degrade expert tractograms by half on the Sørensen-Dice scale, which is normalized between 0 and 1. A Dice of 0.5 commonly reached in the case of the distortions tested in this work corresponds to an overlap of very poor quality in view of the ground truth. It is therefore important to note that the tractogram errors that we are trying to correct here are significant and lead to aberrant results as evidenced by the illustrations of the work.

A limitation of this study is the use of a unique tractography algorithm. However, this one (probabilistic tractography algorithm based on FOD) was chosen in accordance with the state of the art: He et al. (2021) demonstrate, in the case of the retinogeniculate visual pathway, that this algorithm provides the highest overall reconstruction rate and Xie et al. (2020) establish that a higher order model-based tractography algorithm had better performance on identifying true positive structures in comparison to the single-tensor tractography algorithm in the trigeminal nerve. Finally, this choice is confirmed by the recent study conducted on the same dataset used here (Jacquesson et al., 2018a) where all the cranial nerves could be tracked in the contralateral hemisphere of the tumor and with an 87% success rate on the displaced nerves.

### 5.2. Accuracy of Tractogram Filtering

The entropy and FOD methods have shown to be able to provide better filtering compared to the ground truth than the FA-based method. This is probably due to their more global nature. The FA-based method integrates punctual information along the fiber while entropy and FOD methods also integrate contextual information. In the case of FA method, the information is specific to each of the voxels without considering the contextual neighborhood, whereas in the case of the entropy and FOD methods, the information is, respectively, specific to a neighborhood adapted to the size of the nerve and to all the diffusion MR image. In addition, it should be noted that the entropy method—which is a statistical measure of the disorder—makes it possible to introduce an anatomical prior of coherence on the tractogram while the FOD method ensures to guarantee the fit between the fibers of the tractogram and the underlying MR diffusion image.

These differences in the information that is embedded in the filtering approaches along the fiber is crucial. Indeed, when an ROI is misplaced in the cisternal cavity of the nerve, other very proximal bundles can be selected which the FOD and entropy can get rid of thanks to the contextual information from which they benefit. When the ROI is too large, on the other hand, the bundles selected in addition to the nerve of interest can be of very different nature. In this case, only very global information as produced by the FOD makes it possible to remove these bundles. Finally, in the context of a poor parameterization of the FA threshold, the tracing of the cranial nerve can continue for a longer time and in particular in the brainstem where white fibers mingle leading to spurious fibers. In this specific case, only the entropy method makes it possible to track a valid nerve because it encodes the coherence sought by the expert.

However, if the FOD method provides the best filtering results, the new approach developed (entropy) benefits of a reasonable complexity as it only integrates local information, adapted to the size of the nerve. Besides, as it does not depend on the original diffusion MR image, it can be generalized to other fibrous structures. We compared the FOD and entropy methods from a computational time point of view but they are not implemented in the same computer code and the FOD method is parallelized while ours is not yet.

### 5.3. Perspectives and Recommendations

To go further in the automation of the tractography in surgical planning, we plan to build a probabilistic atlas of ROIs by registering and merging the ROI data of the database used in this work (Jacquesson et al., 2018a). Since our filtering approach is able to deal with dilatation and translation of the ROIs, it seems adequate to compensate for the future use of such an ROI atlas and more particularly with possible patient-specific physiological variations, which would not be encoded in the atlas. From this study, especially the results depicted in Figures 6, 8, we are able to identify the challenges linked to the automation of cranial nerve tractography for presurgical planning and make the following recommendations.

Our study demonstrates that filtering makes it possible to correct the distortions resulting from a bad parameterization of the tractography on the tractograms of cranial nerves. More precisely, we deduced from the results of Figure 8 that the tolerated error (with regard to the optimal parameter) is 0.03 for FA, and 2*D*/5 for the size of the ROI (where *D* is the size of the nerve of interest). We make more careful recommendations concerning the ROI translations. Unlike the variation of FA or ROI size, ROI translations tend to truncate the nerves, as shown in Figure 5. The filtering algorithms cannot compensate for this lack of nervous fibers. For this reason, we recommend to limit the translations as much as possible and to anticipate possible misplacement by increasing the size of the ROI. This information will be taken into account to build the ROI atlas.

### 5.4. Application in Neurosurgery

**Figure 12:**
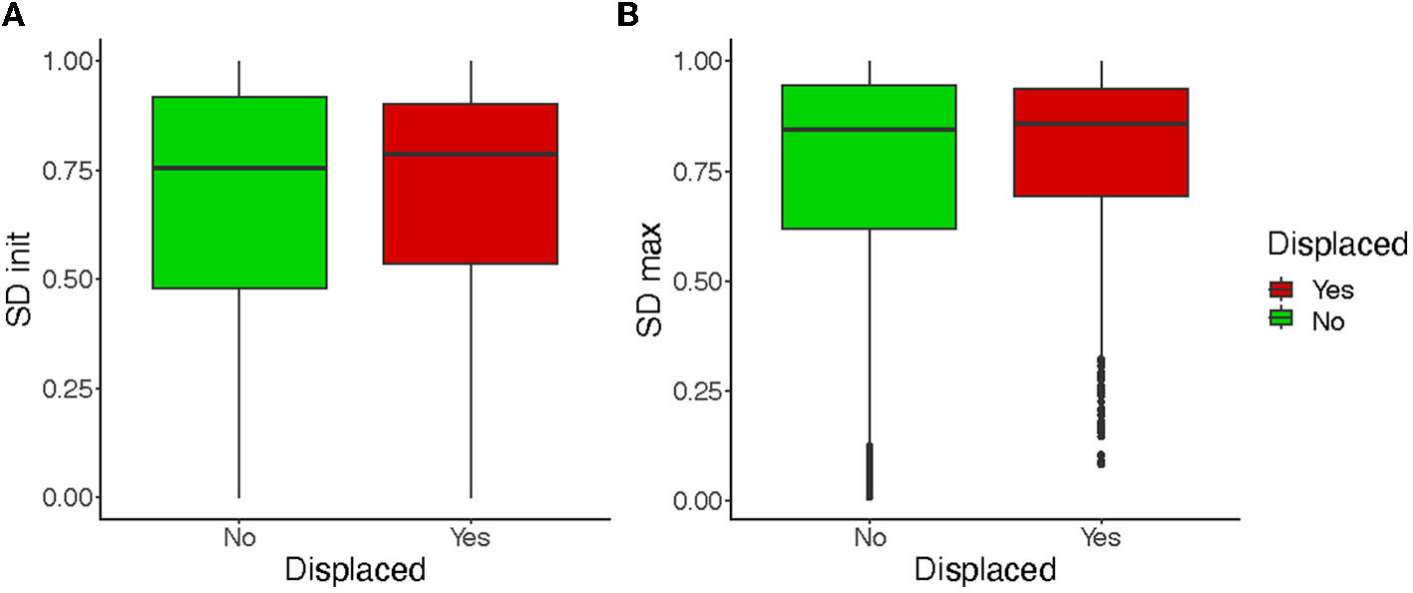
Comparison of the distributions of *SD*_init_ **(A)** and *SD*_max_ **(B)** for displaced and non-displaced nerves. *SD*_max_ is given on average on the basis of all the filtering methods.

The patients included in this study presented tumors of the base of the skull, which displaced cranial nerves (on average, 2 nerves or more were affected by patient). The tractography parameters were adapted by the expert to track those displaced nerves (Jacquesson et al., 2018a). To know whether this displacement impacts in the sensitivity of the tractograms to parameter variation and the effect of the filtering, we consider independently the group of displaced and non-displaced nerves. Figure 12 shows that the quality of the initial tractograms is not statistically different for the nerves displaced than the others (*p* = 0.3) and that the impact of filtering is similar for the two groups of nerves (*p* = 0.22). Filtering appears to be an automatic method for increasing the quality of tractograms for both intact nerves and displaced nerves. This opens up important perspectives for the targeted application in neurosurgery. A such filtering method could be directly implemented in navigation systems or tractography software to provide an accurate tractography with less user-related steps (Jacquesson et al., 2019). Entropy or FOD filtering could also improve the quality of selective brain fiber tracking in brain connectivity studies.

## 6. Conclusion

We have proposed a method to assess the quality of a filtering method applied to a cranial nerve tractogram. The filtering was carried out by an original approach that selects the fibers of the tractogram from a measurement of entropy applied in the neighborhood of each voxel in order to improve the biological precision of the tractogram. This method was compared with two classical other approaches: a method based on FA which integrates only local information and the more recent method based on the FOD which integrates information on the entire diffusion MR image. We show that by removing the fibers considered to be detrimental to the quality of the tractogram given the ground truth, the bias of incorrect parameterization of the tractography is reduced in the filtered datasets and the biological plausibility of the tractograms is improved. Our filtering approach offers filtering performance exceeding the FA approach and similar to the FOD except in the case of too large ROI size. It also offers great possibilities in terms of automation with an optimal threshold that is easier to set and a more modest algorithmic complexity.

## Data Availability Statement

The Entropy filtering code is available in the following GitHub project: https://github.com/megdec/tractography-visualization. It also includes a test dataset. Patient data is not available but may be available on special request and under conditions relating to the protection of personal data. Further inquiries can be directed to the corresponding author.

## Ethics Statement

The studies involving human participants were reviewed and approved by CPP SUD-EST II, Bâtiment Pinel, Groupement Hospitalier Est, 59 Boulevard Pinel 69500 BRON. The patients/participants provided their written informed consent to participate in this study. Written informed consent was obtained from the individual(s) for the publication of any potentially identifiable images or data included in this article.

## Author Contributions

MDec, TJ, and CF contributed to the conception and design of the study. MDec, TP, MV, and MA organized the database and performed the numerical experiments. MA produced the illustrations on the patients. MDes performed the statistical analysis. MDec wrote the first draft of the manuscript. MDec, MDes, TJ, and CF wrote sections of the manuscript. All authors contributed to manuscript revision, read, and approved the submitted version.

## Funding

Support was received from the Servier Institute; the Hospices Civils de Lyon; the French Society of Neurosurgery; the Medtronic, and Nuovo Soldatis Foundations.

## Conflict of Interest

The authors declare that the research was conducted in the absence of any commercial or financial relationships that could be construed as a potential conflict of interest.

## Publisher's Note

All claims expressed in this article are solely those of the authors and do not necessarily represent those of their affiliated organizations, or those of the publisher, the editors and the reviewers. Any product that may be evaluated in this article, or claim that may be made by its manufacturer, is not guaranteed or endorsed by the publisher.
